# Supplementary material for: Storage and Utilization of Glycogen by Mouse Liver during Adaptation to Nutritional Changes Are GLP-1 and PASK Dependent
Source: Nutrients. 2021 Jul 26;13(8):2552. doi: 10.3390/nu13082552 (PMC8399311; doi:10.3390/nu13082552)
Supplement: Supplementary file 1 [file nutrients-13-02552-s001.zip › nutrients-1283028-Supplemantary-Tables.pdf]

Supplemental Table S1. Identification of primers used in the different gene expression assays

REAL-TIME POLYMERASE CHAIN REACTION (SYBR GREEN qPCR)

Gene	Mouse Forward primer	Mouse Reverse primer
Acc (ACC)	5′-CCTCTTCCTGACAAACGAG-3′	5′-TCCATACGCCTGAAACATG-3′
Actb (β-ACTIN)	5′-CTCTCTTCCAGCCTTCCTTC-3′	5′-GGTCTTTACGGATGTCAACG-3′
Chrebp (CHREBP)	5' -CTGGGGACCTAAACAGGAGC-3'	5' -GAAGCCACCCTATAGCTCCC-3'
Cpt1a (CPT1A)	5′-CATGTCAAGCCAGACGAAG-3′	5′-TGGTAGGAGAGCAGCACCT-3′
Fas (FAS)	5′-AAGGCTGGGCTCTATGGATT-3′	5′-GGAGTGAGGCTGGGTTGATA-3′
Foxo1 (FOXO1)	5′-GACAGCCGCGCAAGACCAG-3′	5′-TGAATTCTTCCAGCCCGCC-3′
Glut2 (GLUT2)	5′-TGTGCTGCTGGATAAATTCGCCTG-3′	5′-AACCATGAACCAAGGGATTGGACC-3′
Gys2 (GYS)	5′-ACTGCTTGGGCGTTATCTCTGTG-3′	5′-ATGCCCGCTCCATGCGTA-3′
G6pase(G6Pase)	5′-TTACCAAGACTCCCAGGACTG-3′	5′-GAGCTGTTGCTGTAGTAGTCG-3′
L-pk (L-PK)	5′-TTGCTCTACCGTGAGCCTC-3′	5′-ACCACAATCACCAGATCAC-3′
Lxra (LXRA)	5′-GCCCTGCACGCCTACGT-3′	5′-TAGCATCCGTGGGAACATCA-3′
Mcad (MCAD)	5′-TTCGAAGACGTCAGAGTGC-3′	5′-GCGACTGTAGGTCTGGTTC-3′
Pepck (PEPCK)	5′-CCACAGCTGCAGAACA-3′	5′-GAAGGGTCGCATGGCAAA-3′
Ppargc1α (PGC1α)	5′-ATGTGTCGCCTTCTTGCTCT-3′	5′- ATCTACTGCCTGGGGACCTT-3′
Pparα (PPARα)	5′-TGTTTGTGGCTGCTATAATTT-3′	5′-GCAACTTCTCAATGTAGCCTA-3′
Pparγ (PPARγ)	5′-GTGCCAGTTTGCATCCGTAGA-3′	5′-GGCCAGCATCGTGTAGATGA-3′
Pygl (PYGL)	5′-TGGCAGAAGTGGTGAACAATGAC-3′	5′-CCGTGGAGATCTGCTCCGATA-3′
Scd1 (SCD1)	5′-CTGACCTGAAAGCCCGAAG-3′	5′-GCGTTGAGCACCAGAGTGTA-3′
Sirt1 (SIRT1)	5′-TTGTGAAGCTGTTCGTGGAG-3′	5′-GGCGTGGAGGTTTTTCAGTA-3′
Sirt2 (SIRT2)	5' -AGCCAACCATCTGCCACTAC-3'	5' -CCAGCCCATCGTGTATTCTT-3'
Srebp-1c (SREBP1C)	5′-GGAGCCATGGATTGCACTT-3′	5′-GCTTCCAGAGAGGAGGCCAG-3′

REAL- TIME POLYMERASE CHAIN REACTION (TAQMAN® ASSAY)

Gene	Probe identification (Taqman® Assay)
18s (18S)	Hs99999901_s1
Gck (GCK)	Mm00439129_m1
Gckr (GCKR)	Mm00523328_m1
Pask (PASK)	Mm00435916_m1

Supplemental Table S2. Antibodies and conditions used for western blot assays

Antibody	Host	Manufacturer	Dilution used
Anti-AKT	Rabbit	Cell Signaling, Danvers, MA, USA	1:1000
Anti-GCK	Rabbit	Santa Cruz Biotechnology, California, USA	1:1000
Anti-GCKR	Rabbit	Santa Cruz Biotechnology, California, USA	1:1000
Anti-GLUT2	Mouse	Santa Cruz Biotechnology, California, USA	1:1000
Anti-GYS	Mouse	Santa Cruz Biotechnology, California, USA	1:1000
Anti-Mouse-HRP	Goat	Bethyl Laboratories, Montgomery, USA	1:5000
Anti-phospho AKT1/PKBα (Ser 473)	Mouse	Milipore Iberica, Madrid, Spain	1:1000
Anti-phospho GYS (Ser 641)	Rabbit	Cell Signaling, Danvers, MA, USA	1:1000
Anti-PTEN	Rabbit	Milipore Iberica, Madrid, Spain	1:500
Anti-Rabbit-HRP	Goat	Milipore Iberica, Madrid, Spain	1:5000
