# Supplementary material for: Storage and Utilization of Glycogen by Mouse Liver during Adaptation to Nutritional Changes Are GLP-1 and PASK Dependent
Source: Nutrients. 2021 Jul 26;13(8):2552. doi: 10.3390/nu13082552 (PMC8399311; doi:10.3390/nu13082552)
Supplement: Supplementary file 1 [file nutrients-13-02552-s001.zip › nutrients-1283028-Supplementary-Figures.pdf]

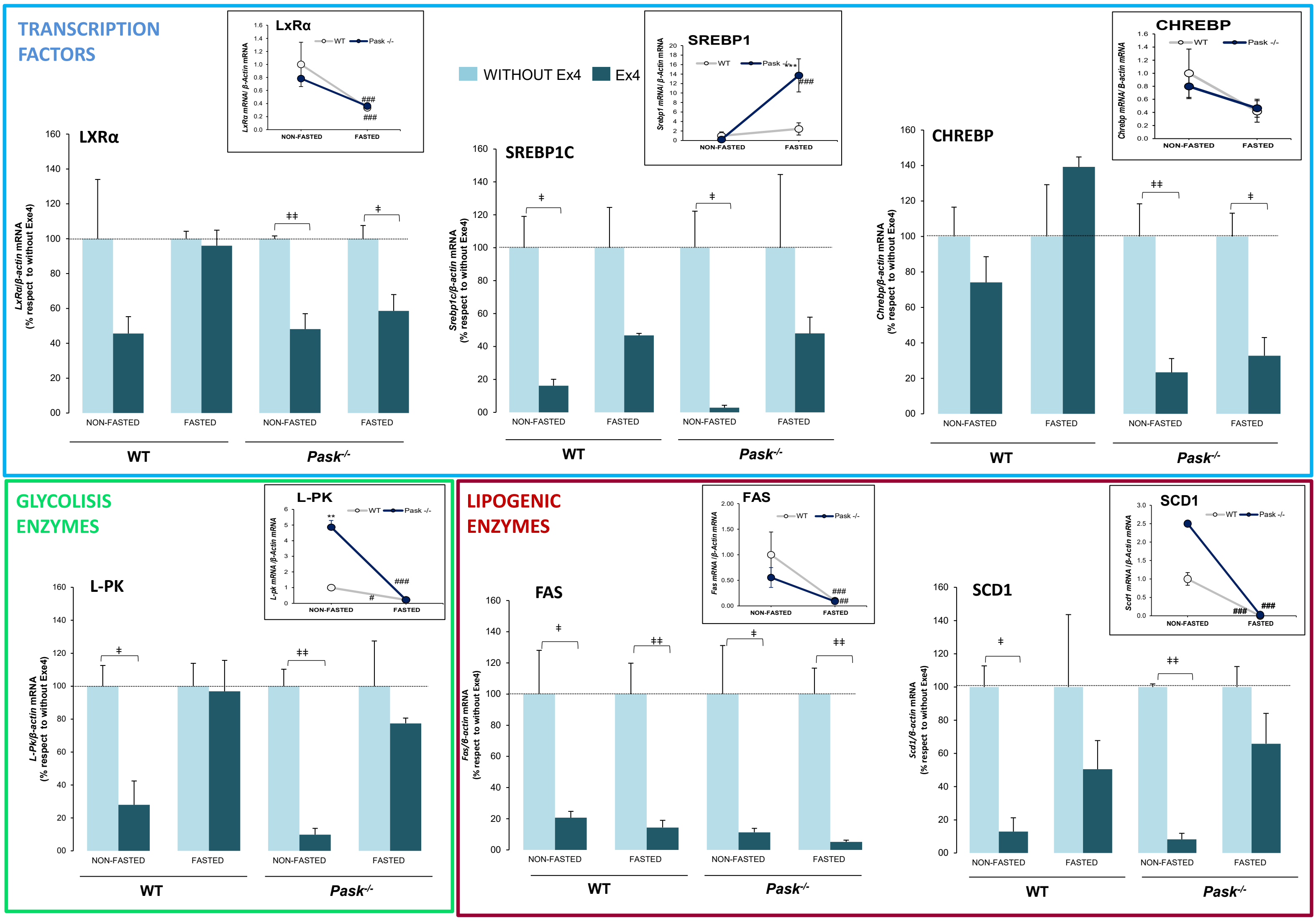

Figure S1. Effect of exendin-4 in the expression of hepatic metabolic genes usually activated by feeding. Real-time PCR was used to analyze the mRNA levels of *Lxrα, Srebp1c, Chrebp*, *L-pk*, *Fas* and *Scd1*. The expressions were measured in the livers of non-fasted (NON-FASTED) and 48-h fasted (FASTED) from wild-type mice (WT) and PASK-deficient mice (*Pask*$^{-/-}$) treated or not for 3 h with exendin-4 (Ex4). The mRNA levels of different genes were normalized by the mRNA of *β-actin* used as housekeeping gene. Bar graphs represent the means ± SEM, the value obtained in each condition in the absence of exendin-4 was taken as 100; n = 3-5 animals per condition. $^{‡}p$ < 0.05; $^{‡‡}p$ < 0.01 vehicle *vs*. exendin-4. Inserts represent the differences on the mRNA expression between WT and *Pask*$^{-/-}$ mice in non-fasted and 48-h fasted states ($^{\#}$p< 0.05; $^{\#\#}p$ < 0.01; $^{\#\#\#}p$ < 0.001 non-fasted *vs* fasted; * p< 0.05 WT *vs Pask*$^{-/-}$)

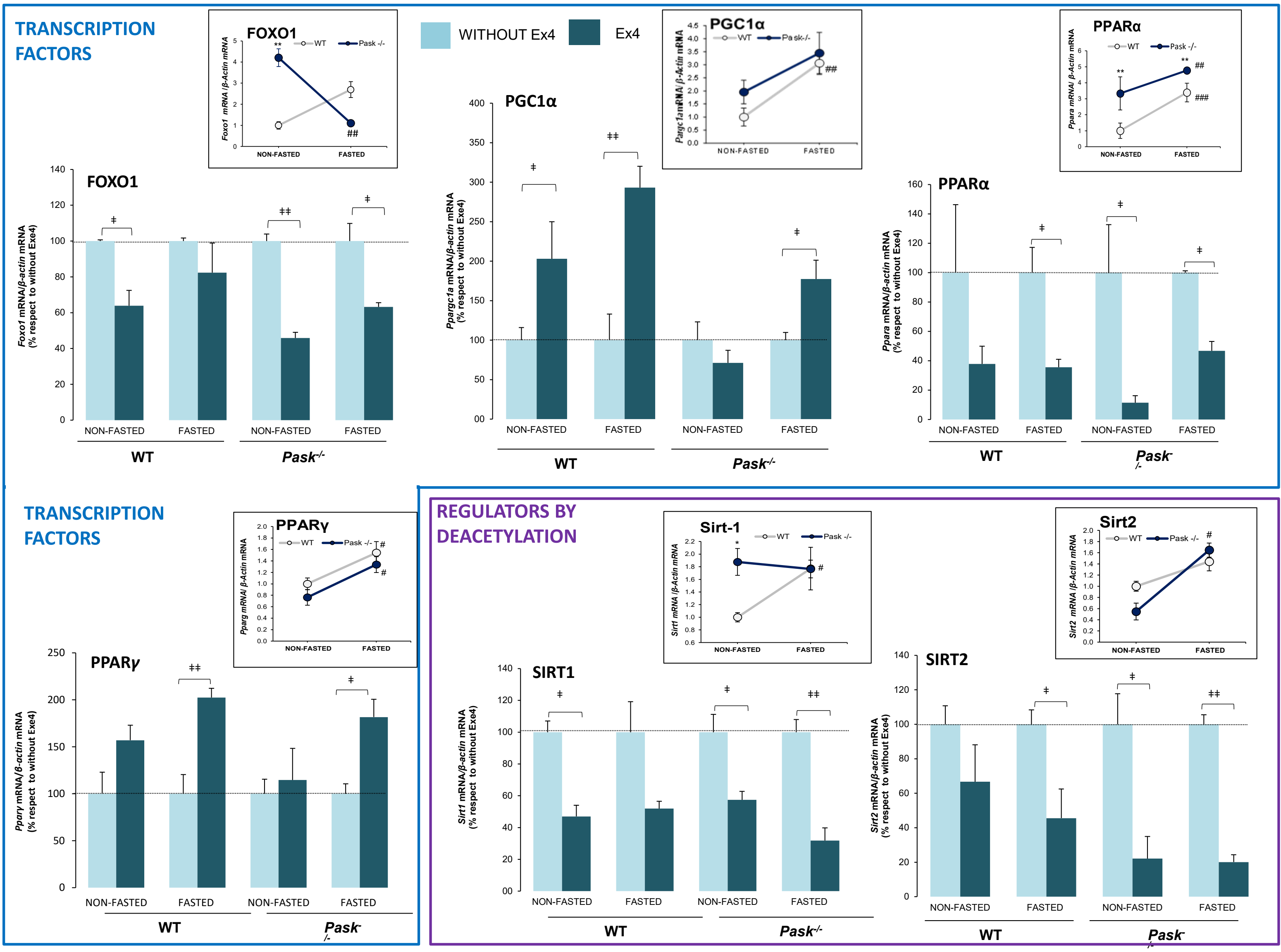

Figure S2. Effect of exendin-4 in the expression of hepatic metabolic genes usually activated by fasting. Real-time PCR was used to analyze the mRNA levels of *Foxo1, Ppargc1α, Pparα*, *Pparγ*, *Sirt1* and *Sirt2*. The expressions were measured in the livers of non-fasted (NON-FASTED) and 48-h fasted (FASTED) liver from wild-type mice (WT) and PASK-deficient mice (*Pask*$^{-/-}$) treated or not for 3 h with exendin-4 (Ex4). The mRNA levels of different genes were normalized by the mRNA of *β-actin* used as housekeeping gene. Bar graphs represent the means ± SEM, the value obtained in each condition in the absence of exendin-4 was taken as 100; n = 3-5 animals per condition. $^{‡}p < 0.05$, $^{‡‡}p < 0.01$ vehicle *vs*. exendin-4. Inserts represent the differences on the mRNA expression between WT and *Pask*$^{-/-}$ mice in non-fasted and 48-h fasted states ($^{\#}p < 0.05$; $^{\#\#}p < 0.01$; $^{\#\#\#}p < 0.001$ non-fasted *vs* fasted; ** p< 0.05 WT *vs Pask*$^{-/-}$)

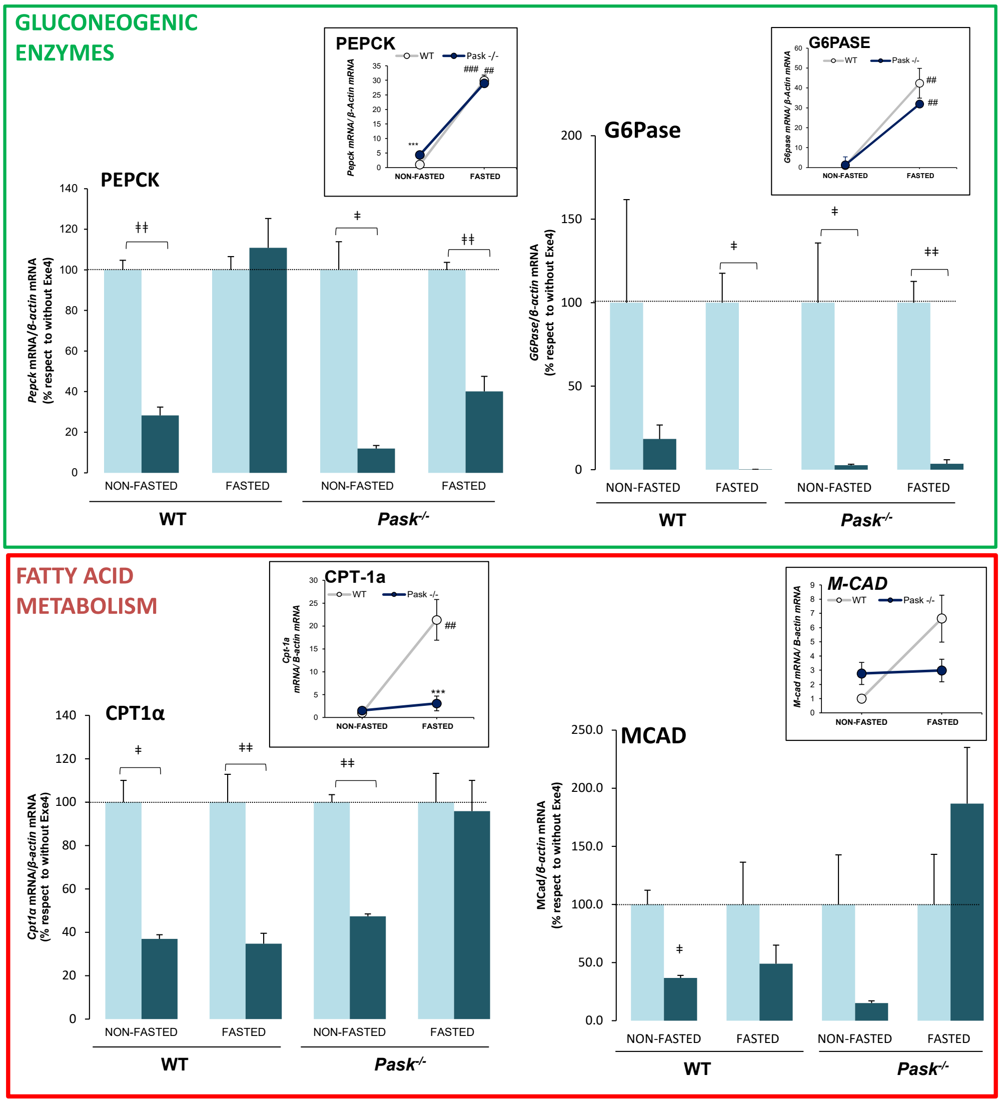

Figure S3. Effect of exendin-4 in the expression of hepatic metabolic genes usually activated by fasting. Real-time PCR was used to analyze the mRNA levels of *G6pase, Pepck, Cpt1* and *Mcad*. The expressions were measured in the livers of non-fasted (NON-FASTED) and 48-h fasted (FASTED) liver from wild-type mice (WT) and PASK-deficient mice (*Pask*$^{-/-}$) treated or not for 3 h with exendin-4 (Ex4). The mRNA levels of different genes were normalized by the mRNA of *β-actin* used as housekeeping gene. Bar graphs represent the means ± SEM, the value obtained in each condition in the absence of exendin-4 was taken as 100; n = 3-5 animals per condition. $^{‡}p < 0.05$, $^{‡‡}p < 0.01$ vehicle *vs*. exendin-4. Inserts represent the differences on the mRNA expression between WT and *Pask*$^{-/-}$ mice in non-fasted and 48-h fasted states ($^{\#}$p< 0.05; $^{\#\#}p < 0.01$; $^{\#\#\#}p < 0.001$ non-fasted *vs* fasted; ** p< 0.05 WT *vs* *Pask*$^{-/-}$)
